# Psychological Impact of the Quarantine-Induced Stress during the Coronavirus (COVID-19) Outbreak among Italian Athletes

**DOI:** 10.3390/ijerph17238867

**Published:** 2020-11-28

**Authors:** Alessandra di Cagno, Andrea Buonsenso, Francesca Baralla, Elisa Grazioli, Giulia Di Martino, Edoardo Lecce, Giuseppe Calcagno, Giovanni Fiorilli

**Affiliations:** 1 Department of Motor, Human and Health Sciences, University of Rome “Foro Italico”, Lauro de Bosis Square, 15, 00197 Rome, Italy; alessandra.dicagno@uniroma4.it (A.d.C.); elisagrazioliphd@gmail.com (E.G.); giulia.dimartino21@gmail.com (G.D.M.); e.lecce1@studenti.uniroma4.it (E.L.); 2 Department of Medicine and Health Sciences, University of Molise, v. De Sanctis 1, 86100 Campobasso, Italy; andreabuonsenso@gmail.com (A.B.); francesca.baralla@unimol.it (F.B.); fiorilli@unimol.it (G.F.)

**Keywords:** pandemic, COVID-19 restrictions, sport, psychological distress, adaptation process

## Abstract

The 2019 Coronavirus (COVID-19) outbreak caused home confinement, as well as training and sport competitions withdrawals. The prolonged inactivity impact, and lack of in-person interactions among teammates-coaches, could negatively affect athletes. Total of 1508 self-selected Italian athletes, 338 children (aged 10.52 ± 1.31), 499 adolescents (aged 14.17 ± 1.13), and 671 adults (aged 27.59 ± 10.73), completed the Impact of Event Scale (IES-8, IES-15, and IES-R, respectively). Differences by gender, type of sport (individual vs. team), and competitive level (elite vs. amateur) were examined. One-way ANOVAs showed, in adults, significant differences between genders for perceived stress impact total score (TS; *p* = 0.017) and avoidance behavior, with higher scores in women (*p* = 0.045). Between individual and team sport, significant differences were found in TS (*p* = 0.038) and hyperarousal (*p* = 0.030), with higher results in individual. Adult elite athletes showed significantly higher scores in hyperarousal (*p* = 0.020) than amateurs. Significant differences were found between gender in adolescents for avoidance (*p* = 0.011), and between competitive levels in children, for intrusion (*p* = 0.020). These evidences may raise awareness on distress effects of COVID-19 lockdown among athletes and suggested applying specific well-being protocols during the activity resumption, considering gender, type of sport, and competitive level.

## 1. Introduction

The Coronavirus disease 2019 (COVID-19) caused a high alert because of its devastating effects on the respiratory system. The high infection risk led the World Health Organization (WHO) to declare the pandemic outbreak [1]. Starting on 9 March 2020, the Italian Prime Minister, after the virus spread, ordered the implementation of physical distancing measures. Schools and many other recreational, cultural, and sports centers must stay closed in order to avoid human-to-human transmission [2,3]. To safeguard the health of athletes and others involved, the Olympics and Paralympics events, as well as other sports competitions at international, national, and regional levels, have been postponed to 2021 or erased. Until 4 May 2020, quarantine measures kept a large number of people in isolation, restricting their activity practice and interactions, with significant psychological effects on individuals and societies. Pellecchia and colleagues [4] suggested that longer quarantine duration, infection fears, inadequate information, financial loss, and stigma represent high-stress factors. Pfefferbaum and North [5], showed that some groups may be more vulnerable than others to pandemic-related psychosocial effects. In fact, extensive researches on mental health have established that after a disaster, most people are resilient and maintain positive outcomes in the complex interplay between risk and vulnerability factors [6,7,8]. Different levels of psychological distress is associated with gender, age education, occupation, and localization. According to Lee [9], there are evident gender differences in both adaptation and responses to potentially traumatic events and life stressors. Kogler and colleagues [10] highlighted a higher resilience and self-manage coping responses to stress in males than women. Moreover, although young people resulted less vulnerable than adults to COVID-19 infection, children and adolescents are more psychologically impacted by the consequent lockdown [11]. It is well-known that sport enhances physical and psychological skills [12,13]. In fact, young athletes, during their sport practice, gradually learn to cope with their emotions, such as anxiety, stress, and anger. In addition, they implement performance subcomponents such as motivation, perceived control, enjoyment, satisfaction [14]. Sport often represents a life goal for elite athletes. For them, training interruption could represent a greater psychological pressure, than for amateurs or young athletes, because of the fear of the disruption to their athletic careers and eventually loss of revenue or sponsorship [15]. Moreover, athletes in this period may experience progressive loss of sport-specific physical fitness, which will result in injury risks when they return to training. There are gendered dimensions to these experiences [16]. Team athletes seem to be less affected by disturbing psychological symptoms. The support of other team members, sharing of responsibility could be beneficial in coping with adversities [17]. Social contact, even if with distancing, could be an effective “buffer” for psychological distress and could help to have a good resilience [18].

Contrariwise, prolonged inactivity, boredom, lack of social interactions with teammates and coaches, may represent stressors; in particular for children and adolescents, adding up to the possible negative effects caused by the pandemic and the interruption of the normal training routines [19]. Sedentary attitude, due to lockdown, led to negative effects on children and adolescents, such as mental swings, depression, a decrease of general health [20]. Recent studies pointed out the protective role of physical activity during the lockdown [21], nevertheless, the long quarantine duration in Italy, compared to other countries, could have affected the psychological development and well-being of children and adolescent athletes [22], as well as of the adult athletes [23]. Considering the different epidemic spread levels that occurred in the different Italian geographical areas [24], it could be newsworthy to know if athletes from different Italian places showed different reactions, even if identic quarantine measures were put in place throughout Italy. Research data are required to develop evidence-driven strategies to reduce adverse psychological effects during the pandemic, helping the policymakers in comprehending problems of different population typologies and to mitigate the effects [25,26]. It is needed to identify high-risk groups, among athletes, to carry out early and efficient intervention [27]. Specifically, it is important to understand the implications of such unusually prolonged exercising absence conditions on athlete health and well-being [22].

In the present research, existing scales of self-perceived stress were used to identify specific categories of experiences in acute response to stressful events. The three versions of the Impact of Event Scale (respectively, IES-8, IES-15, and IES-R) were used to track psychological distress perception among Italian athletes after being exposed to life events, such as sports activities withdrawal. This study aimed to evaluate the acute stress-perception and stress-response reactions to sports activity interruption, due to the quarantine measures applied during COVID-19 outbreaks in Italy, among different aged Italian athletes, considering gender, competitive levels (elite vs. amateur), type of sport (individual vs. team), and geographical areas (North, Centre, South).

The observed data may provide evidence about the emotional-reactions related to the COVID-19 restrictions among Italian athletes, in order to give specific support to overcome the uncomfortable situation, as well as to re-organize optimal training programs for sports activity resumption.

## 2. Materials and Methods

### 2.1. Participants

The participants were recruited through a snowball sampling strategy, using an online survey platform (Google Form—Google, Mountain View, CA, USA). Participants’ inclusion criteria were: age (8 years or more) and affiliation to a national sports federation or associations.

About 5.75% of the participants in the study were excluded because they did not respond or fulfil all the questionnaire items. A total of 1508 self-selected Italian athletes was included in this study: 338 children aged from 8 to 12 years, 499 adolescents aged from 13 to 17 years, 67 adults, from 18 to 67 years respectively. Regarding the geographic area, the sample was divided into the North, Centre, and South of Italy. Athletes were classified by individual (e.g., track and field; gymnastics) or team sports (e.g., basketball, volleyball, and soccer). Based on the competitive level, athletes were divided into elite and amateur athletes. Participants’ characteristics are shown in Table 1.

### 2.2. Procedures

To assess the sports activities’ withdrawal psychological impact, due to the first phase of quarantine in Italy (9 March 2020–18 May 2020), a national web-based survey on athletes was carried out in this period (from the beginning of April to 4 May 2020). The inclusion criteria consist of being a member of a national federation, sports club, and being aged over 8 years.

An informative letter, reporting the main purpose of the study related to the effects of perceived stress following the sports activities’ interruption, was sent to the National Federations and Sports Associations Presidents. The letter included also the link to the online-survey, openly accessible to the nationwide athletes. 

Before the questionnaire, a covering letter explained the nature of the research, including assurance of confidentiality and anonymity. The personal data were collected anonymously through the creation of a personal security code. 

All participants aged over 18 years fulfilled their form after having specified they have read the study’s description and agreed to the terms as described. Electronic informed consent was obtained from all participants via e-mail. For under-aged participants, informed consent was provided by parents or guardians who had to administer the questionnaire to their sons, on behalf of the child.

In the web-survey’s first section, the administered questionnaire aimed to assess socio-demographic factors, such as gender, age, region of residence, type of sport (individual vs. team), and competitive level (elite vs. amateur). 

The second section was focused on the assessment of psychological distress following the sports activity withdrawal. The three different versions of the Impact of Event Scale questionnaire were used, according to the participants’ age. The study was designed following with the Declaration of Helsinki and was approved by the Scientific Technical Committee of the Department of Medicine and Health Sciences, University of Molise (Prot. n. 19/2020).

### 2.3. Screening Questionnaire

The Impact of Event Scale (IES) is a self-administered questionnaire designed to assess the current subjective distress and used in the present survey to evaluate the psychological impact of the sports activity withdrawal due to the COVID-19 quarantine. This event could be considered a potentially traumatic event (PTE), based on individuals’ perception of loss [28].

To assess the psychological distress level, following the sports activity withdrawal, three different databases were used, according to the population age: children underwent the Impact of Event Scale-8 (IES-8) questionnaire [29], Teenagers underwent IES-15 [30], and Adults underwent the Impact of Events Scale-Revised (IES-R) [31].

The Impact of Event Scale-Revised (IES-R) [31], used for participants older than 17 years, includes 22-items, each one with a Likert rating scale from 0 (not at all) to 4 (extremely). The scale evaluates one’s reactions to life events during the last seven days that may cause significant psychological stress in those exposed and possibly leading to a different post-traumatic stress symptom (PTSS). The Total Score (TS) superior to 32 (cut-off), out of a maximum of 88, identifies the amount of distress associated with a specific event. The questionnaire includes three subscales aimed to evaluate the symptoms of intrusion (INT), avoidance (AV), and hyperarousal (HYP) [32]. The subscales scores indicate these different behaviors’ frequencies. INT examines recurring thoughts, images, dreams, and feelings of traumatic experience-related. AV refers to the attempts to remove actively from consciousness thoughts and emotions associated with the traumatic experience; it is related to numbness and dissociation, such as active defensive reaction. HYP responses included symptoms such as anger, irritability, difficulty in concentration, and hypervigilance. The IES-R showed high internal consistency for INT (α: 0.87 to 0.92), AV (α: 0.84 to 0.85), and HYP (α: 0.79 to 0.90). It has also a good test-retest correlation [33]; respectively: INT (0.57 to 0.94), AV (0.51 to 0.89), and HYP (0.59 to 0.92).

Both IES-8 and IES-15 are composed of 8 and 15 items, each with a Likert rating scale from 0 (not at all) to 5 (often). The Impact of Event Scale-15 (IES-15) [30], used for participants aged 13 to 16, measures two main distress’ dimensions: INT and AV. It was showed that adolescents suffering from stress response syndromes have a cut-off score of 30 or higher.

The subscales scores, such as INT and AV, indicate the possibility that these different behaviors occur. It has a good test-retest reliability (0.79 to 0.89), and satisfactory internal consistency (Cronbach’s α = 0.78 to 0.82) [30,31,32,33,34].

The Impact of Event Scale-8 (IES-8) [29], used for participants aged from 8 to 12 years, has a good correlation with the full version (IES-R) and a cut-off score of 17 or higher, that identifies stressed subjects. It has good reliability (0.84) for the whole scale; for the INT and AV subscales, it was 0.91 and 0.83. The IES-8 showed a high internal consistency (Cronbach’s α = 0.78) [35]. The IES-8 was previously used to assess children’s reactions to a potentially traumatic event [36]. The versions of IES used in this research were validated in Italy [37,38].

### 2.4. Statistical Analysis

The SPSS^®^ version 23.0 for Windows [39] was used to analyze the data. Distributions of individual items were assessed, including missing data. A descriptive analysis of the sample was carried out. Categorical variables are presented as frequencies (%) and absolute numbers (N). Continuous values are expressed as mean (M) and standard deviation (±SD). 

The Kolmogorov–Smirnov test was applied, before analysis, to test the normal distribution of data. Because of the non-normal distribution of data, to evaluate differences among IES-8, IES-15, IES-R total score and IES-R subscales’ (INT, AV, and HYP) as dependent variables and gender, Geographic Area (North, Centre, and South of Italy), Type of Sport (individual vs. team), and Competitive Level (elite vs. amateur) as independent variables, a non-parametric statistical approach was used through Kruskal–Wallis test. Post-hoc comparisons were performed using the U test of Mann–Whitney and the Bonferroni alpha level correction was applied. Besides, the internal consistency of the IES-R was evaluated using Cronbach’s α coefficient (α = 0.70). The alpha test level for statistical significance for all variables was set at 0.05.

## 3. Results

Five hundred and forty participants (35.8%), among the 1508 respondents, showed subjective distress symptoms related to training and sport competitions withdrawals during COVID-19 quarantine. 

More than 50% (181) of athletes interviewed among children, 31.68% among adolescents, and 29.81% among adult, obtained an IES-TS higher than the cut-off (Table 2). 

The Kruskal-Wallis analysis, performed on IES-TS, did not show significant differences in children and adolescents for gender, geographic area, type of sport, and competitive level. Significant differences were found between gender for IES-TS (*p* = 0.017) and AV only in adults, with higher result in females (*p* = 0.045). 

Significant differences between individual and team sport for IES-TS (*p* = 0.038) and HYP (*p* = 0.030) were found in adults, with higher scores for individuals. 

The higher competitive level (elite) of athletes showed significantly higher scores in HYP (*p* = 0.020). Referring to adolescents, significant differences between gender for AV (*p* = 0.011), with higher scores in males, were found. 

Although, significant differences between competitive levels for INT (*p* = 0.020), with higher results scores in elite athletes, were found in children.

Means and SD were reported in Table 3.

## 4. Discussion

The COVID-19 outbreak has upset many regular aspects of life, including training routines and sport activity [40]. 

Considering that athletes take up most of their daily life in training and competitions, the COVID-19 quarantine has given rise to feelings of emptiness that may impact mental wellbeing [21], especially of the elite athletes [41,42]. 

The majority of participants in our sample were not greatly disturbed by the quarantine. 

The sports practice, in which athletes usually deal with stressful situations, such as competitive events, leads to achieving skills to manage anxiety and self-control in daily life. The athletes’ repeated exposure to exercise may have led to a stress response system adaptation and a negative cognitive appraisals reduction [43], according to other findings that considered physical activity and stress relationships within improving wellbeing outcomes via exercise [44]. 

Regarding the subjective distress perception (TS), data showed that 35.8% of the participants obtained a higher degree of severity of distress, which is age-related. Especially, more than 50% among children, 32% among adolescents, and 30% among adult athletes resulted in being distressed.

Age and self-perceived stress were significantly interconnected in our results: younger respondents experienced a higher stress level [45]. In a previous study [11], Children seem to have developed peritraumatic distress symptoms compared to the older athletes. The prolonged quarantine, in Italy, may impact especially on vulnerable populations [46]. There is also evidence [47] that psychological distress arises at a young age. Consequently, this study puts close attention to young people’s reactions to quarantine problems, as lockdown and loss of physical activity opportunities and sport behaviors [48]. Young athletes seem to be clingier in their behaviors and daily routines, whereas older athletes react to adverse conditions with anxiety, anger, restlessness, and withdrawal [49]. Several studies identified children’s main psychological reactions during the lockdown: poor sleep, including nightmares, physical discomfort, agitation and inattention, clinginess, and separation problems [11,12,13,14,15,16,17,18,19,20,21,22,23,24,25,26,27,28,29,30,31,32,33,34,35,36,37,38,39,40,41,42,43,44,45,46,47,48]. Sleep quality may be disturbed by high levels of HYP, with distress and anxiety symptomatology [12].

The differences among geographic areas responders were not statistically significant, although different levels of epidemic risks were identified [11,12,13,14,15,16,17,18,19,20,21,22,23,24,25,26,27,28,29,30,31,32,33,34,35,36,37,38,39,40,41,42,43,44,45,46,47,48,49,50]. It was previously stated that participants living in more affected geographic areas showed higher scores of stress, because of quarantine or infection [24,25,26,27,28,29,30,31,32,33,34,35,36,37,38,39,40,41,42,43,44,45,46,47,48,49,50,51]. However, since the quarantine measures have been the same throughout Italy the sample did not show different psychological reactions. Nevertheless, the low participation of North Italy athletes, the most Covid-19 affected area, could indicate that they may not be willing to participate in the questionnaire survey [27].

According to previous findings, significant differences, gender-related on perceived stress, and emotional avoidance-response behaviors were found [52]. Gender differences are evident in adult athletes, regarding adaptation processes and responses to potentially traumatic events and life stressors [53]. Generally, women achieved higher scores on self-perceived stress [54], as well as in the emotional response, and mental health disorders [55]. In relation to adults, gender differences in coping strategies are well-documented [56], and women appear to subjectively experience more stress than men, also with somatoform symptoms [57,58]. Female athletes showed higher AV levels than males, as previous studies have demonstrated [59,60]. Although virtual ones, male athletes could use social contacts to resolve the stressful situation such as the sports activity withdrawal [61]. Shuer and Dietrich [62] found that AV could be a psychological defense to actively remove unpleasant thoughts and situations. Athletes are often familiar to using dissociative strategies to separate life problems from their performance. This approach, defined as “compartmentalization,” could have masked the presence of the psychological symptoms in female athletes [63]. Compared to females, male athletes showed a lower level of emotion-oriented coping styles [64]. 

As previous studies confirmed, male adolescent athletes were more likely to use avoidant coping strategies than females, with several attempts to actively remove thoughts and avoid situations that are reminiscent of the stressful situation [65,66]. Conflicting results on this topic were found in previous studies [30,31,32,33,34,35,36,37,38,39,40,41,42,43,44,45,46,47,48,49,50,51,52,53,54,55,56,57,58,59,60,61,62,63,64,65,66,67]. AV is a typical athletes’ reaction associated with a long duration and potential for repeated traumatic events [62]. The sports activity withdrawal, perceived as uncontrollable and stressful, might favor avoidant coping strategies, tending to distance themselves from stressful situations, and engage in substitute activities [68]. Consequently, it could be advisable to engage more adolescents in enjoyable activities and problem-solving skills to prevent an excess of AV-related behaviors [49]. 

Regarding the different competitive levels, the participation of high-performance youth sport could lead to several negative outcomes that limit the focus on the sport’s potential benefits [69]. Intrapersonal and interpersonal challenges focus on winning, pressures from coaches and parents, enhance the level of stress of young Elite athletes more than amateurs [70]. Elite children athletes showed a high level of intrusive behaviors. Intrusive thoughts are typical reactions to the uncontrollable acute stage of traumatic events. The activity interruption could have enhanced the psychological peritraumatic response with intrusive experiences, such as frequent reminiscence of their activity. It is possible that young athletes are not still able to oscillate between the intrusive and avoidant states, as adult athletes, since these oscillations decrease in magnitude the perception of the traumatic event [71]. 

Significant differences were also found among adult athletes in the stress perception (IES-TS) between the individual and team sport: individual performers showed a higher level of distress than team sport athletes. The different psychological profiles, between the individual and team sport athletes, highlighted that team sport athletes have better control of anxiety, optimism, and resilience than individual sport athletes [72]. Evidence also suggested that Individual sport athletes showed higher personal responsibility and perseverance for their training and competition results than team sport athletes [73]. The withdrawal of training and competition caused more stress and tension in individual athletes worried about the lockdown, which stopped their preparation. Conversely, team sports involve distributing rules and sharing responsibility [74,75]. According to previous studies [17,18], frequent contacts with their colleagues and risk-sharing have represented a protective factor against quarantine isolation consequences and uncertainty of the future training restart. The present data also showed that those who are individual athletes had the highest HYP symptoms than those who are team athletes. Individual athletes are closely linked with specific symptoms of HYP or AV behaviors, that potentially compromise athletic performance, especially when exposed to situations similar to the event.

Elite-level athletes showed significantly higher scores in HYP. The athletes who have a strong athletic identity, such as individual sport athletes or high-level athletes, may experience greater emotional symptoms following a traumatic event [76]. Moreover, the impossibility of maintaining their social networks and the reduced physical activity has been associated with anxiety and this kind of distress [77]. Clemente-Suárez [78] showed that the perception of uncertainty and lack of control *di per sè* may increase different psychopathological reactions such as anxiety or even depression. Moreover, the inability to compete may have influenced the emotional response of adaptation to stress with HYP [42,43,44,45,46,47,48,49,50,51,52,53,54,55,56,57,58,59,60,61,62,63,64,65,66,67,68,69,70,71,72,73,74,75,76,77,78,79] significantly more in elite athletes than amateurs. These findings are in accordance with other researches on elite sports data that reported symptomatology associated with HYP [80], and sleep alteration [81]. Moreover the feeling on athlete’s career paused or concluded, lost revenue, home-based training programs highly affected the elite athletes [82]. 

### Limitations

The first limitation of the study was the voluntary participation in an online self-report evaluation. Consequently, this sample was a non-probability sample.

Another limitation regards the lack of information about the health status related to COVID-19 infection of participants, concerning specific comorbidity and/or pre-existing chronic disease. 

No causal inferences have been analyzed and discussed.

The study refers only to the participants’ psychological acute reaction to the lockdown problem. Further studies could assess the potential chronic consequences, at the end of the pandemic.

## 5. Conclusions

The COVID-19 pandemic has had, and will continue to have effects on people’s physical and mental well-being all around the world, also on athletes and sporting world.

Children, adolescent, and adult athletes demonstrated different stress reactions to their sport activity break. Nevertheless, if adequately supported by social connections, including their teammates and coaches, and their sport environment, they can successfully address the distress condition. 

Increasing communications, playing collaborative games, and increasing physical activity could be a good solution to alleviate emotional and psychological distress due to this long quarantine period. 

Moreover, to avoid psychological discomforts due to this new situation, it could be advisable that the preventive measures are not perceived as compulsory or, such as individual freedom loss, nevertheless interiorized as a common behavioral choice. So, it will be feasible to restart without risks once these preventive interventions are relaxed. Since children and adolescents, in particular, tend to worry more when they do not clearly understand what is happening around them, providing age-appropriate information about the reasons and needs of the sports withdrawal could reduce anxiety and reassure them [83].

Along these lines, social interactions, which are essential to athletes’ psychological well-being and resilience, will be safe and allowed. Clear communication and a good level of knowledge on the preventive measures could have protective effects on pandemic’s psychological consequences in athletes and particularly in children and adolescents [25,26,27]. Consequently, the athletes’ involvement in managing directives and precautionary measures may facilitate adaptive stress response and better adherence [84,85].

This report provides useful information for professionals of sports organizations to organize future athletes’ sports activities in rapid evolution situations.

## Figures and Tables

**Table 1:** Sample characteristics.

Variable	Children	Adolescents	Adults
	*n* (%)	*n* (%)	*n* (%)
Total	338 (100.0)	499 (100)	671 (100.0)
Age (Mean ± SD)	10.52 ± 1.31	14.17 ± 1.13	27.59 ± 10.73
Gender			
Male	172 (50.89)	242 (48.5)	374 (55.74)
Female	166 (49.11)	257 (51.5)	297 (44.26)
Geographic Area			
North	35 (10.36)	48 (9.62)	178 (26.53)
Centre	183 (54.14)	244 (48.9)	269 (40.09)
South	120 (35.5)	207 (41.48)	224 (33.38)
Type of Sport			
Individual	157 (46.45)	252 (50.5)	312 (46.5)
Team	181 (53.55)	247 (49.5)	359 (53.5)
Competitive Level			
Amateurs	153 (45.27)	330 (66.13)	303 (45.16)
Elite	185 (54.73)	169 (33.87)	368 (54.84)

**Table 2:** IES-total score (IES-TS) questionnaires (mean ± SD).

Total Score	Children		Adolescents		Adults	
	*n* (%)	Mean ± SD	*n* (%)	Mean ± SD	*n* (%)	Mean ± SD
Lower than the cut-off	157 (46.45%)	10.66 ± 3.46	340 (68.32%)	17.89 ± 6.93	471 (70.19%)	19.11 ± 7.39
Higher than the cut-off	181 (53.55%)	23.02 ± 4.93	159 (31.68%)	38.25 ± 7.13	200 (29.81%)	43.37 ± 9.84

Children cut-off: ≥17; adolescents cut-off: ≥30; adults cut-off: ≥33.

**Table 3:** Mean ± SD of total score and subscales values.

Variables	Total Score	Intrusion	Avoidance	Hyperarousal
	Mean ± SD	Mean ± SD	Mean ± SD	Mean ± SD
Children	23.02 ± 4.93	12.84 ± 3.72	10.18 ± 4.13	-
Gender				
Male	23.28 ± 5.23	13.41 ± 3.88	9.87 ± 4.20	-
Female	22.75 ± 4.61	12.26 ± 3.48	10.49 ± 4.05	-
Geographic Area				
North	22.75 ± 3.94	12.62 ± 2.85	10.12 ± 3.52	-
Centre	23.32 ± 5.07	12.82 ± 3.89	10.50 ± 4.20	-
South	22.66 ± 4.96	12.94 ± 3.70	9.72 ± 4.16	-
Type of Sport				
Individual	22.71 ± 4.57	12.56 ± 3.69	10.15 ± 4.23	-
Team	23.31 ± 5.24	13.11 ± 3.75	10.20 ± 4.05	-
Competitive Level				
Amateurs	22.44 ± 5.01	12.07 ± 3.71	10.37 ± 3.77	-
Elite	23.49 ± 4.83	13.47 ± 3.63 *	10.02 ± 4.41	-
Adolescents	38.25 ± 7.13	17.91 ± 6.66	20.34 ± 5.39	-
Gender				
Male	39.03 ± 7.78	17.53 ± 7.34	21.50 ± 5.29 *	-
Female	37.60 ± 6.53	18.20 ± 6.07	19.40 ± 5.31	-
Geographic Area				
North	35.00 ± 4.37	16.33 ± 8.26	18.67 ± 6.61	-
Centre	38.51 ± 7.02	17.64 ± 6.47	20.87 ± 5.53	-
South	38.67 ± 7.62	18.57 ± 6.51	20.10 ± 4.91	-
Type of Sport				
Individual	37.68 ± 6.59	17.88 ± 6.22	19.80 ± 5.19	-
Team	38.84 ± 7.66	17.93 ± 7.13	20.91 ± 5.57	-
Competitive Level				
Amateurs	38.40 ± 7.06	17.63 ± 6.66	20.77 ± 5.27	-
Elite	37.94 ± 7.31	18.44 ± 6.68	19.50 ± 5.57	-
Adults	43.37 ± 9.84	17.44 ± 4.26	14.81 ± 4.13	11.12 ± 3.68
Gender				
Male	42.07 ±9.85	16.83 ± 4.20	14.37 ± 4.41	10.87 ± 3.57
Female	44.54 ± 9.74 *	17.99 ± 4.25	15.21 ± 3.84 *	11.34 ± 3.78
Geographic Area				
North	43.80 ± 10.26	18.35 ± 4.33	14.22 ± 3.82	11.24 ± 3.82
Centre	42.70 ± 9.76	17.28 ± 4.13	14.63 ± 4.38	10.79 ± 3.31
South	43.98 ± 9.76	17.02 ± 4.35	15.48 ± 3.96	11.49 ± 4.06
Type of Sport				
Individual	45.20 ± 11.09 *	18.05 ± 4.67	15.43 ± 4.58	11.72 ± 4.00 *
Team	41.65 ± 8.19	16.86 ± 3.76	14.24 ± 3.59	10.55 ± 3.27
Competitive Level				
Amateurs	43.21 ±8.83	17.53 ± 3.94	15.16 ± 3.87	10.52 ± 3.34
Elite	43.50 ± 10.66	17.37 ± 4.52	14.51 ± 4.34	11.62 ± 3.89 *

* Significantly difference (*p* < 0.05).
